# Searching for Abiotic Tolerant and Biotic Stress Resistant Wild Lentils for Introgression Breeding Through Predictive Characterization

**DOI:** 10.3389/fpls.2022.817849

**Published:** 2022-03-03

**Authors:** María Luisa Rubio Teso, Carlos Lara-Romero, Diego Rubiales, Mauricio Parra-Quijano, José M. Iriondo

**Affiliations:** ^1^ECOEVO Group, Área de Biodiversidad y Conservación, Universidad Rey Juan Carlos, Madrid, Spain; ^2^Instituto de Agricultura Sostenible, Spanish National Research Council, Córdoba, Spain; ^3^Facultad de Ciencias Agrarias, Universidad Nacional de Colombia Sede Bogotá, Bogotá, Colombia

**Keywords:** crop wild relatives, predictive characterization, ecogeographic land characterization maps, drought tolerance, salinity tolerance, waterlogging tolerance, rust resistance, broomrape resistance

## Abstract

Crop wild relatives are species related to cultivated plants, whose populations have evolved in natural conditions and confer them valuable adaptive genetic diversity, that can be used in introgression breeding programs. Targeting four wild lentil taxa in Europe, we applied the predictive characterization approach through the filtering method to identify populations potentially tolerant to drought, salinity, and waterlogging. In parallel, the calibration method was applied to select wild populations potentially resistant to lentil rust and broomrape, using, respectively, 351 and 204 accessions evaluated for these diseases. An ecogeographic land characterization map was used to incorporate potential genetic diversity of adaptive value. We identified 13, 1, 21, and 30 populations potentially tolerant to drought, soil salinity, waterlogging, or resistance to rust, respectively. The models targeting broomrape resistance did not adjust well and thus, we were not able to select any population regarding this trait. The systematic use of predictive characterization techniques may boost the efficiency of introgression breeding programs by increasing the chances of collecting the most appropriate populations for the desired traits. However, these populations must still be experimentally tested to confirm the predictions.

## Introduction

In the last century, the advances in plant breeding in search of the most productive and nutritional cultivars have allowed feeding millions of people (Khush, 2001). However, food security is menaced by the decrease in the diversity of crop species and the genetically uniform crop cultivars resulting from the breeding for higher yields (Esquinas-Alcázar, 2005; Khoury et al., 2014, 2021). Climate change is affecting crop production and food security, with different impacts depending on the area of the world and the economic status of the country (Rosenzweig and Parry, 1994; Challinor et al., 2009, 2014; Wheeler and von Braun, 2013; Rosenzweig et al., 2014). The reduction of genetic diversity in crops has made modern crop cultivars more vulnerable to biotic and abiotic stresses which are further aggravated by climate change (Heal et al., 2004; Massawe et al., 2016). Additionally, the development of adapted cultivars in many crops is constrained by this reduced genetic variation (Dempewolf et al., 2017). The adaptation of agriculture to climate change is imperative (Howden et al., 2007), and acquiring the traits to make crops tolerant to biotic or abiotic stresses is fundamental for food sustainability (Newton et al., 2011).

Crop wild relatives are plant species closely related to crops that can, relatively easily, transfer genetic material to them (Heywood et al., 2007). Because of their evolution under natural conditions with non-directed selective pressures, crop wild relatives are adapted to current environmental conditions and are, thus, a valuable source of genetic diversity of adaptive value and traits (Hawtin et al., 1996; Brozynska et al., 2016). Crop wild relatives have gained relevance for breeding in the last decades (Hajjar and Hodgkin, 2007; Jump et al., 2009) and their application as donors of useful traits is widely documented (e.g., Warschefsky et al., 2014; Choudhary et al., 2017). Nevertheless, their incorporation in breeding programs has been limited for several reasons. These include the potential incorporation of undesired traits during the breeding trials (Walley and Moore, 2015; Prohens et al., 2017 and references therein), the difficulty of generating favorable combinations of genes during the hybridization (Sano, 1993), and the impossibility of genetically characterizing every single wild population in the search for traits, being necessary to carefully select which accessions should be screened (Baute et al., 2015). However, given the great value of crop wild relatives as adaptive trait donors, their utilization is now considered a relevant pathway to incorporate genetic diversity (Guarino and Lobell, 2011; Greene and Warburton, 2017; Montenegro de Wit, 2017; Egan et al., 2018; Coyne et al., 2020; Kilian et al., 2020) and there have been considerable advances to overcome the above-mentioned limitations and enhance their use (Ford-Lloyd et al., 2011; Zhang et al., 2017; Zhang and Batley, 2020). Some of them include the creation of new elite materials from crop wild relatives, ready to be used for breeding through the “introgressiomics” approach (Prohens et al., 2017), the use of biotechnology and genomic tools (Baute et al., 2015; Walley and Moore, 2015; Pratap et al., 2021) that make available a rapid gene targeting, or the speed breeding approach that has been successfully applied in different crops (Watson et al., 2018).

The use of ecogeographical and climatic data, together with ecological modeling is also arising as a promising tool, helping in the selection of wild germplasm or populations, both for its use in breeding and for prioritizing their conservation (Castañeda-Álvarez et al., 2016). Similarly, the Focused Identification of Germplasm Strategy (FIGS) (Mackay and Street, 2004) and predictive characterization techniques (Thormann et al., 2014a,^2016^) have been developed to select subsets of landraces and crop wild relative populations with higher probabilities of containing the desired trait than if randomly selected. These techniques can be applied following two approaches: the “environmental filtering” method and the “calibration” method (Thormann et al., 2014a). Considering that the operation of different selective pressures will result in diverging genetic diversity of adaptive value, the environmental filtering method presumes that populations inhabiting certain areas with particular environmental characteristics will probably be better adapted to those conditions than other populations (e.g., populations inhabiting drier areas would be more tolerant to drought). The environmental filtering method does not require the previous characterization of the populations, just information about the environmental conditions existing in each population site, which can be obtained through environmental data available in global and national databases. This method involves the generation of ecogeographic land characterization maps (Parra-Quijano et al., 2012b) that are used as a proxy to maximize the genetic diversity of the subset. Once the populations are ecogeographically and environmentally characterized, guided by literature or expert advice, environmental thresholds that may determine the presence of the desired trait are set, filtering in this way the subset of populations most likely to possess the trait (Thormann et al., 2014a). On the other hand, the calibration method, based on the same premises, needs a set of populations previously evaluated for a given trait (e.g., a set of populations with known resistance or sensitivity to a particular disease). These are used to train a model that will predict, in a given set of non-evaluated populations, those which most likely have the desired attribute. A recent example proving the usefulness of the calibration method is the successful prediction of the acyanogenic status of *Trifolium repens* L. populations after a worldwide screening (García Sánchez et al., 2019).

Cultivated lentil (*Lens culinaris* ssp. *culinaris* Medik.) was probably originated in the Fertile Crescent Area (South-Western Asia and the Mediterranean) (Smartt, 1984; Cokkizgin and Shtaya, 2013), during the Neolithic period, having as probable ancestor *L. culinaris* ssp. *orientalis* (Boiss.) Ponert (Chahota et al., 2019). Its cultivation and domestication probably date back to the late 5th or early 4th millennia BC and it has been described as probably being the most ancient crop among grain legumes (Sandhu and Singh, 2007). Lentils are commonly used in different cropping systems (Rawal and Bausal, 2019) and are traditionally used as a rotational crop which can increase their value as grain legumes if their potential as pre-crop cultivation is leveraged (Preissel et al., 2015). According to the latest FAO reports (FAO, 2020), lentil is cultivated in 44 countries, Jordan is the country with the highest yield (3,400 kg/ha calculated for 2019), and Canada is the country with the largest harvested area (close to 1.5 million hectares in 2019). In 2019, the harvested area in Europe was 137,161 hectares, which resulted in a total of about 125 thousand tons and a calculated yield of 909.6 kg/ha (FAO, 2020). Erskine (2009) reported an average production of 42 thousand tons per year for Europe in the period 2002–2006, which accounted for just 1.1% of global lentil production. However, in the last decade, there has been a large increase in lentil production (an average of 72.9 thousand tons per year in the 2010–2014 period and 173.25 thousand tons in 2015–2019) (FAO, 2020), which highlights a growing trend and increasing interest in its cultivation in Europe.

The lentil yield is affected by a series of biotic (rust, anthracnose, powdery mildew, sclerotinia stem rot or broomrape, among others) (Chen et al., 2009) and abiotic (drought, cold, salinity, or waterlogging, among others) stresses (Andrews and McKenzie, 2007; Kumar et al., 2014; Smýkal et al., 2015; Lake and Sadras, 2019). Biotic and abiotic stresses are affected by climate change and are likely to interact, which might cause higher damages to plants (Challinor et al., 2009). Additionally, the cultivated lentil is reported to contain a narrow genetic base (Fratini et al., 2004; Rawal and Bausal, 2019) and in the short history of breeding this crop, cultivated varieties or landraces have been, precisely, the main source of genetic variation for lentil crop improvement (Materne and McNeil, 2007).

Wild relatives of lentil possess valuable genes conferring resistance and tolerance to biotic and abiotic stresses (Gupta et al., 2011) and recent studies have reported promising results in the use of lentil wild relatives for breeding purposes (i.e., lentil rust, powdery mildew, or fusarium wilt resistance and drought and cold tolerance) (Mohar et al., 2020; Asghar et al., 2021). Other works point to the identification of novel genes or alleles involved in overcoming salinity tolerance and incorporating them into commercial lentils, as the path to increase productivity (Dissanayake et al., 2021), so the exploitation of genetic resources of lentil wild relatives gains special interest. The crossability among the different species of lentils has been widely studied, placing *Lens culinaris* ssp. *culinaris*, *L. culinaris* ssp. *orientalis* and *L. culinaris* ssp. *odemensis* (Ladiz.) into the primary gene pool, *L. nigricans* (M. Bieb.) Godr. and *L. ervoides* (Brign.) Grande into the secondary genepool and *L. lammotei* Czefr. into the tertiary gene pool although in the latter, its placement in the secondary gene pool is under discussion [see Kumar et al. (2014) and references therein]. The crossability with species that are not in the primary gene pool is more difficult, but possible with the help of embryo rescue and hormone treatment (Fratini et al., 2004; Cubero et al., 2009; Tullu et al., 2013; Rawal and Bausal, 2019).

The aim of our study was to screen the variability of natural populations of the crop wild relatives of lentil and to select small subsets of populations that are more likely to contain, under different ecological contexts and considering potential genetic diversity, adaptations to drought, high soil salinity, waterlogging and resistance to rust [*Uromyces vicia-fabae* (Pers.) Schröt] and broomrape [*Orobanche crenata* Forsk.], some of the most important abiotic and biotic factors affecting lentil production. These subsets could then be evaluated for the corresponding traits and used in trait introgression breeding. We posed that it is possible to select populations potentially tolerant or resistant to the targeted traits, based on the ecological range of their distribution. Furthermore, we also posed that the ecogeographic information associated with each population will serve as explanatory variables to train models that successfully project the resistance to lentil rust and broomrape in non-evaluated populations. Because this study was developed under the framework of the Farmer’s Pride project^1^, a European H2020 project focused on the establishment of a network of genetic reserves of crop wild relatives and landraces in Europe and Turkey, the geographic scope of the study was delimited to these areas.

## Materials and Methods

### Study Taxa

*Lens* Mill. genus belongs to the Fabaceae family and contains four species and four subspecies according to the more recent accepted taxonomy (Ferguson et al., 2000). All of them are annual herbs (Castroviejo and Pascual, 1999) and are naturally distributed in the Mediterranean European countries (Euro+Med Plant Database, 2006). The four species are self-pollinating and diploid (2n = 14), with similar karyotypes (Ladizinsky, 1979; Erskine et al., 2016; Nair, 2019) although with potentially higher karyotypic variation than expected (Cubero et al., 2009).

We considered in our study the three species and the two subspecies of *Lens* naturally occurring in Europe and Turkey. These are *L. ervoides*, *L. nigricans*, and *L. lammotei*, as well as *L. culinaris* ssp. *orientalis* and *L. culinaris* ssp. *odemensis*.

### Distribution Data

*Lens* taxa distribution data were extracted from a high-quality georeferencing occurrence database of crop wild relative populations in Europe and Turkey generated for the Farmer’s Pride project^1^. This database contains more than 3 million records for 616 prioritized taxa in Europe and Turkey and is the largest database of crop wild relatives built up to date (Rubio Teso et al., 2020). The generation of such database involved the downloading of records of targeted taxa from GBIF (GBIF.org, 2021) and Genesys^2^ repositories, using the packages rgbif (Chamberlain and Boettiger, 2017) and genesysr (Obreza, 2019) in the R environment (R Core Team, 2020). The raw data downloaded from these sources were further cleaned and filtered eliminating geographically non-accurate records, those dated before 1950, removing duplicates and records falling in urban areas, water bodies, or permanent ice or snow according to the ESA CCI Land Cover Project (2017). Finally, records of the same taxa found within a 1 km radius were also removed, assuming they belonged to the same population. Further information and details about the generation and characteristics of this database can be found in Rubio Teso et al. (2020).

### Generation of an Ecogeographic Land Characterization Map

Ecogeographic land characterization maps (ELC maps) are useful tools to represent potential adaptive scenarios, built on the combination of different bioclimatic, edaphic, and geophysic variables characterizing a territory (Parra-Quijano et al., 2012a). In this study, we generated an ELC map for Europe and Turkey based on the occurrence of *Lens* sp. populations, as a proxy of the different adaptive scenarios to which *Lens* populations may be subjected. The different ecogeographic categories obtained with the ELC map were used as an additional criterion in the selection of populations potentially adapted to the abiotic stresses. By maximizing the number of different ecogeographic categories in the selected populations, the background genetic diversity of the subset is likely to be increased, as well as the probability of gathering cases of convergent selection of the same trait through different evolutionary pathways.

The selection of variables explaining lentil taxa distribution, and thus of importance for the generation of the ELC map, was carried out using a modified R script developed for the *SelecVar* tool of CAPFITOGEN3 (Parra-Quijano, 2020). The analysis included ecogeographic variables with available data classified in three components: 65 bioclimatic variables, 35 edaphic variables, and 18 geophysic variables, and also included latitude and longitude, all available in CAPFITOGEN3 local mode (Parra-Quijano, 2020) (Supplementary Material 1). Variables’ data were extracted at 2.5 arc-min resolution (around 5 km × 5 km) for each population, according to their geographical coordinates. The R script extracts the ecogeographic variables from the occurrence sites and assesses the importance of each variable in explaining the distribution of the study populations (Parra-Quijano et al., 2020). It estimates variable importance according to the Random Forest Classification (RF) machine learning algorithm and detects redundant variables through bivariate correlation analysis. RF categorizes variable importance according to their higher mean decrease accuracy (MDA) values (Cutler et al., 2007). The top fifteen bioclimatic and edaphic and geophysic variables were ordered according to their MDA value and correlated variables within the same group were removed (Pearson correlation coefficient > | 0.5| and *p* < 0.05) following Garcia et al. (2017).

The selected variables were then used for the generation of an ELC map following a modified R script of the *ELCmapas* tool of CAPFITOGEN3 (Parra-Quijano, 2020). The map resolution was 2.5 arc-min. The “elbow” method was chosen for the determination of the optimal number of the ecogeographic clustering, allowing a maximum of six clusters per group of variables. This method, which is recommended for large territories (Parra-Quijano et al., 2020), determines the cut-off points based on the decrease in the sum of the intra-group squares (Ketchen and Shook, 1996). It reaches the optimal number of groups when the decrease in the sum of squares in a range of *n* and *n* + 1 group is less than 50% (Parra-Quijano et al., 2020).

Using the ELC map, ecogeographic categories were extracted for each population using a modified R script of the *Representa tool* of CAPFTOGEN3 (Parra-Quijano, 2020) and incorporated into the master table of population occurrences along with the bioclimatic, edaphic, and geophysics information. All the analyses regarding the generation of ELC maps and subsequent data management were performed using the R 3.6.3 version of the R environment (R Core Team, 2020) and scripts downloaded and adapted from CAPFITOGEN3 website^3^.

### Predictive Characterization

Targeted abiotic stresses were drought, salinity, and waterlogging. The search for their tolerance in wild relative populations of lentils was performed using the environmental filtering method (Thormann et al., 2014a,b). Targeted biotic stresses were lentil rust (*Uromyces vicia-fabae*) and broomrape (*Orobanche crenata*). Resistance to both diseases, potentially found in wild populations, was modeled using the calibration method (Thormann et al., 2014a,b).

#### Calculation of Aridity Indexes, Soil Textures, and Delimitation of Saline and Non-saline Soils

The De Martonne aridity index (De Martonne, 1926) was obtained for each record as a proxy to estimate the drought stress experienced by the targeted populations. De Martonne aridity index (I_*ar*_DM) was calculated as follows:


$$I_{a\,r}\,D\,M=\frac{P}{T+10}$$

where “P” is the Annual Mean Precipitation, “T” the Annual Mean Temperature, and 10 is a constant to avoid negative values.

Drought stress during the flowering season is reported to severely affect plant development (Kazan and Lyons, 2016). Thus, a De Martonne Aridity Index adapted to the flowering season (I_*ar*_DM_*f*_) was generated by calculating the mean of the monthly De Martonne Aridity Index (I_*ar*_DM_*m*_) for the flowering period (March, April, May, and June). The monthly De Martonne Aridity Index (I_*ar*_DM_*m*_) and the Flowering season De Martonne index (I_*ar*_DM_*f*_) were calculated for each population with the following expressions:


$$I_{a\,r}\,D\,M_{m}=12\,\frac{P}{T_{m}+10}$$

$$I_{a\,r}\,D\,M_{f}=\frac{I_{a\,r}\,D\,M_{m\,a\,r\,c\,h}+I_{a\,r}\,D\,M_{a\,p\,r\,i\,l}+I_{a\,r}\,D\,M_{m\,a\,y}+I_{a\,r}\,D\,M_{j\,u\,n\,e}}{4}$$

Calculated aridity indexes were included in the master table (Table 1).

**TABLE 1:** Classification of areas according to De Martonne Aridity index (De Martonne, 1926).

De Martonne classification—Aridity	De Martonne Index Value	Classification
	0 ≤ I_ar_DM<5	Deserts. Extremely arid
	5 ≤ I_ar_DM<10	Semi-desert. Arid
	10 ≤ I_ar_DM<20	Drought Mediterranean countries. Semi-arid
	20 ≤ I_ar_DM<30	Sub-humid
	30 ≤ I_ar_DM<60	Humid
	I_ar_DM ≥ 60	Per-humid

**Soil texture classification**	**Superficial soil content combinations**	**Soil Texture**

	If Clay ≥ 40%, Sand ≤ 45% and Silt < 40%	Clay
	If Clay ≥ 40% and Silt ≥ 40%	Silty Clay
	If Clay ≥ 35% and Sand > 45%	Sandy Clay
	If Clay ≥ 27% and < 40% and Sand ≤ 20	Silty Clay Loam
	Different combinations	Other

**Soil salinity classification (based on soil conductivity)**	**Conductivity (dS/m)**	**Type of soil and effect on crop plants**

	0–2	Non-saline: Salinity effects negligible
	2–4	Slightly saline: Yields of sensitive crops may be restricted
	4–8	Moderately saline: Yields of many crops are restricted
	8–16	Strongly saline: Only tolerant crops yield satisfactorily
	>16	Very strongly saline: Only a few very tolerant crops yield satisfactorily

*Classification of soil textures according to their content in clay, silt, and sand (USDA, 2020). Classification of soils according to the conductivity of the saturation extract (dS/m) and their effects on crops (Abrol et al., 1988).*

Superficial soil contents in clay, silt, and sand of population occurrence sites, obtained from the Harmonized World Soil Database (Fischer et al., 2008), through the CAPFITOGEN3 tools (Parra-Quijano, 2020), were used to categorize soil texture into Clay, Silty-Clay, Sandy-Clay, and Silty-Clay-Loam or Other (Table 1) using the Soil Texture Calculator (USDA, 2020). The resulting categories were then added to the master data table.

Regarding soil salinity, sites were classified according to soil conductivity, into five categories, from non-saline to very strongly saline soils (Abrol et al., 1988; Table 1).

#### Environmental Filtering Method—Abiotic Stress Analyses

Populations more likely to be tolerant to drought, soil salinity, and waterlogging were selected using an R script adapted from Van Etten et al. (2011) (Supplementary Material 2). Ecogeographic categories of the ELC map of Europe and Turkey were taken into account so they were proportionally represented in the selected subset. The purpose was to generate three subsets with a maximum of 30 populations per trait, to be proposed for seed collecting and subsequent evaluation for the target traits in characterization and evaluation trials.

##### Drought Tolerance

Populations with an Annual De Martonne Aridity (I_*ar*_DM) below 15 (mid-low semi-arid areas) were selected as a first subset. This initial selection contained less than 30 populations. From this subset, those populations with Flowering De Martonne Aridity indices (I_*ar*_DM_*f*_) below 15 were selected and ranked according to I_*ar*_DM_*f*_, from most to least arid conditions during the flowering season.

##### Salinity Tolerance

Since most crops reduce their yields under saline conditions (soil conductivity above 4 dS/m) (Panta et al., 2014; Zörb et al., 2018), a first subset targeted moderately saline soils or higher (i.e., soil conductivity above 4 dS/m). However, none of the populations of our dataset occurred under conditions that matched this criterion. Thus, a second subset was generated that selected populations occurring in slightly saline soils (soil conductivity > 2 dS/m < 4 dS/M).

##### Waterlogging Resistance

A first subset was generated by selecting populations inhabiting areas where limited water drainage was expected according to their soil texture (Clay, Silty Clay, Sandy Clay, or Silty Clay Loam). Subsequently, from this subset, a second selection was performed, identifying populations occurring in mid-high subhumid areas or more humid, according to their Annual De Martonne Aridity Indices.

#### Calibration Method—Biotic Stress Analyses

To train the models a database with evaluation information on resistance to lentil rust and broomrape for *Lens* sp. accessions was used. The database contained 419 georeferenced populations distributed worldwide that were evaluated using the Disease Severity Rating (DSr), 351 of them assessed for resistance to lentil rust (mean of 4 years’ field trials) (Supplementary Material 3) and 204 for resistance to broomrape (mean of 3 years’ field trials) (Rubiales, unpublished results). A basic description of the contents of the database is shown in Supplementary Material 4.

The binarization of levels of expression of both traits from quantitative values in a continuous range (DSr, 0–100) into qualitative values (resistant, sensitive), was approached by classifying as resistant the accessions with the lowest DSr values, i.e., those located in the first decile of the distribution. This criterion was chosen to ensure maximum levels of resistance in the predicted subset. The binarized levels of expression (0 = susceptible; 1 = resistant) were used as the dependent variables. The same ecogeographical variables that were previously used for the generation of the ELC map were used as explanatory variables.

The calibration process was performed using a modified R script developed for the *Modela* tool of CAPFITOGEN3 (Parra-Quijano, 2020), based on the Biomod2 package (Thuiller et al., 2020). In this case, the R 3.1.2 version of the R environment was used, as recommended by Capfitogen developers (Parra-Quijano pers. comm.). Accessions classified as resistant to lentil rust or broomrape were used as “presence” data and sensitive accessions as “absence” data. Presences and absences were given the same weight independently of their number, the original balance of presences/absences was kept and 100% of the presence data was used to obtain the models. The True Statistic Skill value (TSS) (Allouche et al., 2006) was used to test the performance of nine algorithms: GLM—Generalized Linear Models (Nelder and Wedderburn, 1972), GAM—Generalized Additive Models (Hastie and Tibshirani, 1986), GBM—Generalized Boosting Models (Friedman, 2001, 2002), CTA—Classification Tree Analysis (Breiman et al., 1984), Artificial Neural Networks—ANN (Ripley, 1996), Flexible Discriminant Analysis—FDA (Hastie et al., 1994), Multivariate Adaptive Regression Splines—MARS (Friedman, 1991), Random Forest—RF (Breiman, 2001) and Surface Range Envelopes—SRE [similar to Bioclim (Busby, 1991)]. TSS is currently one of the most widely used evaluators for model performance, as it is not affected by the prevalence of the data (the proportion of sites in which the species is recorded, in our case described as resistant) (Allouche et al., 2006). One hundred runs (models) were performed per each algorithm, using 75% of the data as “training” data—used to calibrate the models—, and 25% of the data as “testing” data, that is to evaluate each model. Variable importance for the models for each algorithm was determined with 100 permutations and ranked on a 0–1 scale (0: no influence in the model; 1: total influence in the model). Subsequently, the mean values of TSS of the 100 runs for each algorithm were calculated and the three algorithms with the highest mean TSS values were compared. Based on the index provided by Thuiller et al. (2010), the performances of the models according to the TSS values were classified into five categories: Fail or null (0 > TSS < 0.2), Poor (0.2 > TSS < 0.4), Fair (0.4 > TSS < 0.6), Good (0.6 > TSS < 0.8) and Excellent or High (0.8 > TSS < 1). From the selected algorithm, the best model (i.e., the run with the highest TSS) was chosen as the predictor model and projected on the non-evaluated populations. Targeted wild populations were then classified as resistant or sensitive according to the cutoff suitability value given by the selected model. Populations with suitability values (ranked from 0 to 1,000) lower than the cutoff value of the model were classified as sensitive and those with suitability values higher than the cutoff value as resistant. From the latter and aiming to provide a manageable set of populations for breeding purposes, the 30 populations with the highest suitability values were selected as the subset with the highest probabilities of being resistant to lentil rust or broomrape.

## Results

### Distribution Data

**FIGURE 1:**
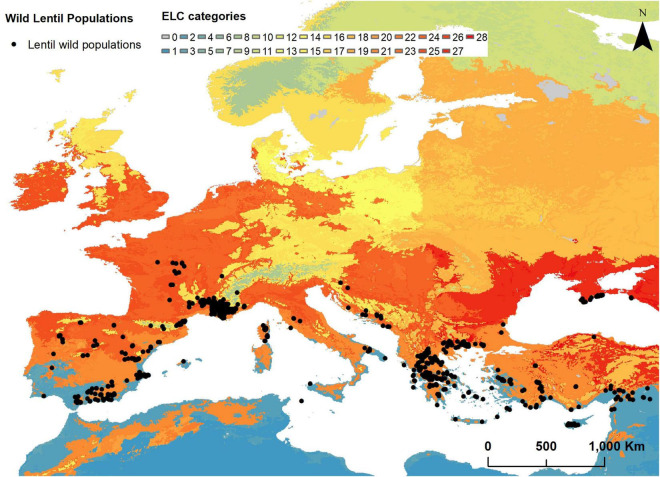
Ecogeographic land characterization map of the wild relatives of lentils in Europe and Turkey (2.5 arc-min resolution) and distribution of wild relatives of lentils in Europe.

The subset with *Lens* wild relatives extracted from the database generated in the Farmer’s Pride project contained 624 populations (Supplementary Material 5). From these, 105 were obtained from the Genesys database, which means that additionally to its probable *in situ* occurrence, are also *ex situ* preserved in gene banks. These entries are identified by Farmer’s Pride Identifier codes starting with “GE”. Populations belong to four different taxa and are distributed in 14 countries covering almost all the European countries in the Mediterranean Basin (Figure 1). The taxon with the highest number of records is *L. nigricans* with 443 populations found in 12 countries, followed by *L. ervoides* which has 145 populations in nine countries. *L. lamottei* and *L. culinaris* ssp. *orientalis* have 29 and 7 populations, respectively, found in three countries each. The countries with the highest number of populations recorded are Greece (173), France (165), and Spain (128).

The subspecies *L. culinaris* ssp. *odemensis* was not included in the analysis due to a lack of high-quality occurrence data.

### Ecogeographic Analyses

#### Selection of Variables for the Ecogeographic Land Characterization Map

The 15 variables per component with the highest MDA values (Supplementary Material 6) were checked for high values of correlation between each other and, as a result, reduced to eight variables. Bivariate correlations for each component are shown in Supplementary Material 7. Hence, in the bioclimatic component, all variables were highly correlated to the one with the highest MDA values (annual mean temperature). In the edaphic component, four non-correlated variables were selected: bulk density (fine earth) of topsoil, topsoil available soil water capacity until a wilting point, topsoil total exchangeable bases, and topsoil sand fraction. Finally, in the geophysic component, three non-correlated variables were selected: annual solar radiation, December solar radiation and longitude.

#### Generation of Ecogeographic Land Characterization Map

The eight selected ecogeographic variables were used for the generation of the ELC map. ELC categories classified as “0” or “NA” were not considered for subsequent analyses, due to the lack of ecogeographic information. The resulting ELC map (Figure 1) has 28 different ecogeographic categories or units, grouped into four bioclimatic, three edaphic, and three geophysic clusters. Variable values per each ecogeographic category are shown in Supplementary Material 8.

#### Ecogeographic Characterization of the Targeted Populations

The extraction of bioclimatic, edaphic, and geophysic variables information assigned values to 619 of the 624 populations under analysis. *L. nigricans* had five populations classified into categories with no data (“0” or “NA”) due to the lack of ecogeographic data in their locations and thus were excluded from subsequent analyses. Populations of wild lentils were distributed in 13 of the 28 categories of the ELC map. *L. nigricans* was the species represented with the highest number of ELC categories (12), followed by *L. ervoides* (9). *L. lamottei* and *L. culinaris* ssp. *orientalis* populations were distributed across three ELC categories (Table 2).

**TABLE 2:** Ecogeographic information of the targeted populations.

	No. pop.	No. ELC cat.	No. semi-arid pop (I_*ar*_DM)	No. semi-arid pop. (I_*ar*_DM_*f*_)	No. pop. soil with clay texture
*L. culinaris* ssp. *orientalis*	7	3	0	0	4
*L. ervoides*	145	9	12	32	17
*L. lamottei*	29	3	12	10	1
*L. nigricans*	438	12	54	40	47
Totals	619	13*	78	82	69

*Number of populations per taxon, number of different ecogeographic categories in which these populations are found, number of populations classified as semi-arid according to Annual (I_ar_DM) or Flowering (I_ar_DM_f_) De Martonne Index values, and number of populations in clayey soils.*

*Totals for No. ELC cat. are the different ELC categories in which the overall populations are found.

Seventy-eight populations were classified as semi-arid according to the Annual De Martonne Aridity Index, but none were classified as arid or extremely arid. *L. culinaris* ssp. *orientalis* did not have any populations classified as semi-arid. According to the Flowering De Martonne Aridity Index, 82 populations were classified as semi-arid, and, again, no populations were found in drier areas. *L. culinaris* ssp. *orientalis* did not have any population in this category either. Concerning soil texture, 69 were classified in Clay soils, whereas no populations were classified with Silty Clay, Sandy Clay, or Silty Clay Loam textures (Table 2).

### Predictive Characterization

We obtained three subsets of lentil wild relative populations that may contain adaptations to tolerate the targeted abiotic stresses and one subset potentially resistant to lentil rust. A complete database with all the ecogeographical information and the results on predictive characterization for each population is found in Supplementary Material 5.

#### Environmental Filtering Method—Abiotic Stress Analyses

##### Drought Tolerance

**FIGURE 2:**
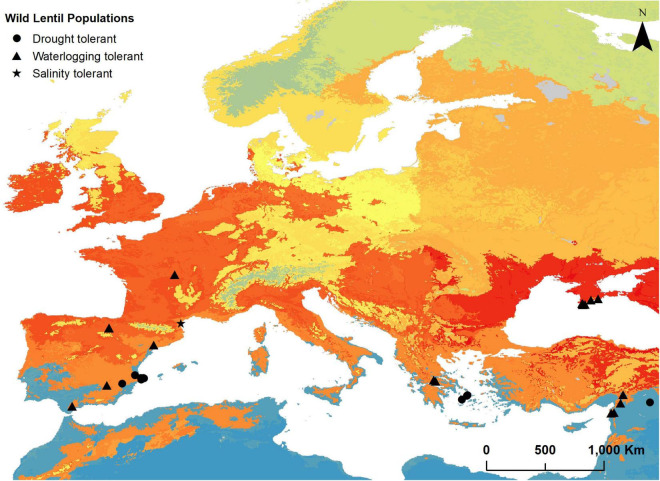
Location of populations of wild relatives of lentils in Europe and Turkey potentially tolerant to drought (black circles), to salinity (black star), and to waterlogging (black triangle). All populations were selected through the environmental filtering method of the predictive characterization technique. Locations are depicted on an ecogeographic land characterization map generated for these taxa to illustrate the potentially different adaptation landscapes.

Thirteen populations belonging to three taxa (*L. nigricans*, *L. lamottei*, and *L. ervoides*) and distributed in Greece (3), Spain (9), and Turkey (1) were selected as potentially tolerant to drought (I_*ar*_DM and I_*ar*_DM_*f*_ below 15). Only one ELC category (ELC category 2) is represented in this subset. Figure 2 shows the location of these populations and Supplementary Material 9 provides complete identification and location details of the populations along with the aridity indices values.

##### Salinity Tolerance

No populations were found to occur in moderately saline (4–8 dS/m soil conductivity) or more saline soils. One population (*L. lamottei*) is in slightly saline soil (soil conductivity above 2 and below 4 dS/m). This population is found in France (Figure 2) and is assigned to ELC category 21. Detailed information on the identification and location of the population can be found in Supplementary Material 9.

##### Waterlogging Tolerance

From the targeted soil texture categories (Clay, Silty Clay, Sandy Clay, or Silty Clay Loam), we only found wild lentil populations in clayey soils (69 populations). Subsequent criterion (I_*ar*_DM > 25, mid-high subhumid areas or more humid) narrowed the selection to 21 populations. These populations belonged to the four taxa, are found in France (1), Greece (2), Spain (6), Turkey (4), and Ukraine (8) (Figure 2), and belong to four different ELC categories. Further details on the location of these populations, Annual De Martonne Aridity Indices, and ELC categories are shown in Supplementary Material 9.

#### Calibration Method—Biotic Stress Analyses

The database with evaluation data for resistance to lentil rust contained 351 accessions whose DSr ranged from 0 (totally resistant) to 94.4 (almost totally sensitive). The binarization of data following the first decile criterion selected 33 accessions as resistant, with DSr values lower than 25. The rest of them were classified as sensitive (318).

Average performances of all models in the algorithms ranged from Null (SRE) to Fair (MARS, FDA, CTA, GLM, RF, and GBM) (Supplementary Material 10). From the algorithms classified as “fair”, the best three were GBM, RF, and GLM with very similar mean TSS values (0.588, 0.58, and 0.54, respectively). Thus, variables’ contribution to the model was assessed (Table 3). All the variables contributed to the best GBM algorithm (TSS value 0.828) giving the higher importance to longitude as an explanatory variable (0.168), followed by the topsoil water capacity until wilting point (0.09) and annual solar radiation (0.086). The random forest best model (TSS value 0.828) gave similar importance to the variables. Finally, the GLM best model (TSS value 0.879) only selected the longitude (0.836) and the topsoil water capacity (0.163) as contributors to the model. As a model-based essentially on longitude is not biologically meaningful from a mechanistic perspective, we excluded this option, and based on the similar variable contribution found in GBM and RF, these two approaches were considered. Subsequently, the highest TSS value criterion was used and the best model of the GBM algorithm was selected as a predictor and projected.

**TABLE 3:** Variable importance given by the best run of the selected algorithms modeling the resistance of wild lentils to rust.

Variable	Model		
	GBM	Random Forest	GLM
Longitude	0.168	0.152	0.836
Topsoil available soil water capacity until wilting point	0.09	0.073	0.163
Annual solar radiation	0.086	0.1	0
Annual Mean Temperature	0.037	0.042	0
Solar radiation December	0.03	0.049	0
Bulk density (fine earth) of topsoil	0.024	0.1	0
Topsoil total exchangeable bases	0.02	0.017	0
Topsoil sand fraction	0.011	0.018	0

*The algorithm selected for projection is in bold.*

**FIGURE 3:**
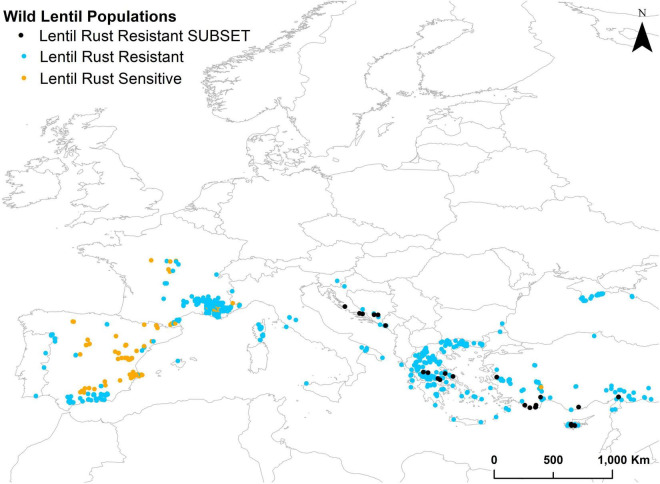
Location of projected lentil rust sensitive (orange dots) and resistant (blue dots) wild lentil populations. The subset of selected lentil rust-resistant (black dots) wild lentil populations.

A cutoff suitability value of 106 was obtained for the best model of the GBM algorithm. When this model was projected to the non-evaluated wild populations it identified 529 populations as potentially resistant to lentil rust (Figure 3). From these, the first 30 with the highest suitability values (248–295) were selected to constitute the subset for screening for lentil rust resistance. These 30 populations belong to *L. ervoides* (21 populations) and *L. nigricans* (9 populations) and are distributed in six countries: Bosnia and Herzegovina (3) Croatia (4), Cyprus (6) Greece (6), Montenegro (1), and Turkey (10) (Figure 3 and Table 4). They represent five of the 13 ELC categories in which the taxa are distributed, although more than a half of them (17 populations) occur in ELC category 21. Detailed information on these populations can be found in Supplementary Material 11.

**TABLE 4:** Countries and taxa corresponding to the subset of 30 populations of wild relatives of lentils with higher probabilities of showing resistance to lentil rust.

Country	Taxon	No. populations	ELC categories
Bosnia and Herzegovina	*L. ervoides*	2	24, 25
	*L. nigricans*	1	25
Croatia	*L. ervoides*	2	21, 24
	*L. nigricans*	2	21, 24
Cyprus	*L. ervoides*	4	21
	*L. nigricans*	2	21
Greece	*L. ervoides*	3	21, 22
	*L. nigricans*	3	21, 22
Montenegro	*L. ervoides*	1	24
Turkey	*L. ervoides*	9	2, 21
	*L. nigricans*	1	21

*The number of populations per taxa and number of Ecogeographic Land Characterization (ELC) categories in which they are distributed.*

All algorithms assayed to model broomrape resistance resulted in a very poor performance (All TSS values < 0.2). Cutoff values different from the ones initially set to define resistant individuals (first decile with the lowest DSr values) were also tested (first 17, 35, and 50% of the data with the lowest DSr values), but they did not improve the fit of the models. Consequently, no projections were made for broomrape resistance.

#### Targeted Trait Overlapping in Selected Subset Populations

None of the populations were simultaneously selected in the four subsets. However, there are some populations’ coincidences between the biotic and abiotic selections. Four populations selected in the drought-tolerant subset were also classified as potentially resistant to lentil rust: three populations of *L. nigricans* in Greece (identifiers in the occurrence database generated for the Farmer’s Pride project: ID_6954734, ID_6954730, and ID_6954729) and one population of *L. ervoides* in Turkey (ID_6953173). In addition, the population of *L. lammotei* selected in France as potentially tolerant to salinity was also classified as potentially resistant to lentil rust (ID_6953872). Finally, 19 populations belonging to the waterlogging tolerant subset were also classified as potentially resistant to lentil rust: two populations of *L. culinaris* ssp. *orientalis*, one in Ukraine (ID_6952604) and one in Greece (ID_6952438); nine populations of *L. ervoides*—one in Greece (ID_6953356), four in Ukraine (ID_6953026, ID_6953338, ID_6953339, ID_6953347) and four in Turkey (GE_222085, GE_222062, GE_222075, GE_222086); one population of *L. lammotei* in Spain (ID_6953903) and finally seven populations of *L. nigricans*, three of them in Spain (ID_6954642, ID_6954569, ID_6954618), one in France (ID_6954460) and three in Ukraine (ID_6954246, ID_6954619, ID_6954710). It is worth mentioning that the four *L. ervoides* populations in the Turkey subset as waterlogging tolerant and classified as potentially resistant to lentil rust, are not only potentially accessible to users in their *in situ* locations—according to our high-quality database—but also have stored accessions in gene banks.

Supplementary Material 5 provides the values obtained for each trait in each population and the target trait overlapping in the indicated populations.

## Discussion

In this study we identified four subsets of populations of wild relatives of lentils, targeting different abiotic and biotic stresses affecting cultivated lentils. These subsets are more likely to be tolerant to drought, salinity, waterlogging, or resistant to lentil rust than randomly chosen populations. However, the modeling algorithms used were not able to satisfactorily identify a subset of populations more likely to be resistant to broomrape. We discuss below the benefits and limitations of our approach, providing arguments that support the use of predictive characterization.

### Ecogeographic Analyses

The variables selected through the objective (non-directed) analyses (annual mean temperature, bulk density, water capacity until wilting point, exchangeable bases, topsoil sand fraction, annual solar radiation, December solar radiation and longitude) make sense when interpreted in the light of the ecology of *Lens* species and their origin. Lentil is reported to be sensitive to acid soils and waterlogging if compared to other grain legumes (Andrews and McKenzie, 2007) and also drought and heat are constraints to obtaining high lentil yields (Rawal and Bausal, 2019). Wild species usually prefer alkaline, stony, or sandy soils (Castroviejo and Pascual, 1999) and thus, the annual mean temperature, the presence of exchangeable bases, the bulk density, sand fraction agree as explanatory variables for their distribution. Furthermore, as in most cultivated plants, solar radiation is known to be a key element in lentil yield in association with other factors (e.g., sowing time, rainfall) (Andrews and McKenzie, 2007). Thus, the plant efficiency in the interception of solar radiation has been shown to be positively related to biomass and yield in lentils (Habib et al., 2021). Finally, the probable origin of lentils in the Fertile Crescent and the Mediterranean (Smartt, 1984; Cokkizgin and Shtaya, 2013) and the East-West distribution of the wild species in the Mediterranean (Ladizinsky, 1979; Ladizinsky et al., 1983, 1984 and references therein), support the selection of the longitude as an explanatory variable.

Ecogeographic approaches are considered a resource to select different populations with potentially useful traits (Hodgkin and Hajjar, 2008), based on the assumption that different environmental conditions will differently shape the genetic diversity and will provide potential adaptations to populations inhabiting distinct environmental sites (Parra-Quijano et al., 2012b). The generated ELC map considered the most important variables for lentil wild relative distribution and covered the whole geographic scope of the project (Europe and Turkey). Given that wild lentil populations are not naturally distributed through the whole range of this territory, finding them in 13 of the 28 ecogeographical categories denotes a wide ecological range of these species. Such wide ELC representation across the wild lentil populations may entail a broad genetic diversity of adaptive value that can boost the potential benefits of using predictive characterization techniques. Other studies using ecogeographic approaches, such as the one by Mezghani et al. (2019) with wild relatives of carrots in Tunisia, also found a great diversification in the ecogeographic distribution of the populations within and across taxa, which they linked to traits of potential interest for breeding. In the same line, Kantar et al. (2015) also reported the presence of *Helianthus* wild species in diverse ecogeographic clusters, finding many of them in extreme environments that allow direct efforts in the selection of populations for abiotic stress tolerance breeding.

### Environmental Filtering Method—Abiotic Stress Analyses

The relatively low number of populations found in semi-arid sites during the flowering period agrees with the literature that highlights that many legume species, including lentils, are severely affected by drought during this period (Andrews and McKenzie, 2007). The occurrence of lentil wild relative populations across a relatively wide range of water availability conditions (from semi-arid to almost sub-humid) suggests that populations living in the most arid conditions may have experienced adaptations to drought. The final subset of 13 populations, obtained by further filtering those simultaneously occurring in sites with De Martonne Annual and Flowering Indexes below 15, provides a more manageable fraction of populations to be handled by breeders, who could select from these those better fitting their particular pre-breeding programs.

Hamdi and Erskine (1996) consider that drought tolerance may not always be related to populations inhabiting arid sites and advocate field trials to confirm the tolerance. This implies that drought-tolerant lentils may be present in other environments and express this trait through phenotypic plasticity responses. These considerations may be sound but do not undermine the fact that drought tolerance is likely to be found, as a result of natural selection, in sites under high water limitation. Consequently, we think our results are trustworthy in the sense that the probability of finding drought tolerance in the given subset is likely to be significantly higher than if the same number of populations were randomly selected from the distribution area. The likelihood of identifying drought-tolerant populations through this approach is further supported by the fact that wild lentil species have shown a wider genetic diversity associated with drought tolerance than cultivated varieties, especially in *L. ervoides* (Singh et al., 2016) and *L. nigricans* (Coyne et al., 2020), and that experimental testing of drought tolerance of wild and cultivated peas found some wild pea accessions better adapted to drought than cultivated pea, as well as ecogeographical patterns associated to aridity gradients (Naim-feil et al., 2017). Additionally, lentils have other strategies to overcome drought, such as drought escape (advance or delay of the flowering time) (Shrestha et al., 2009), which can show a more intense response in semi-arid sites. Finally, other works with legume species have successfully selected drought-tolerant accessions of *Vicia* species through the calibration method of the FIGS approach (Khazaei et al., 2013; Bari et al., 2016). In any case, it is clear that the drought adaptation of the selected populations should be experimentally confirmed.

As for other traits, the identification of genetic variation for salinity tolerance is the first step prior to breeding for salinity tolerant genotypes in lentils (Maher et al., 2003). The predictive characterization method directs the selection of germplasm to be screened, aiming to increase the probabilities of finding the desired trait. Yet, there are few studies of predictive characterization targeting salinity tolerance. Thormann et al. (2016) and Garcia et al. (2017) generated subsets of populations through this method but did not experimentally evaluate them for this trait. In contrast to the number of populations selected for other traits, only one population (*L. lamottei* in slightly saline soil, located in France) was selected for salinity tolerance. Yet, it is a significant result since lentil is one of the most sensitive crops to soil salinity (Ashraf and Waheed, 1990; Yadav et al., 2009), not being able to grow even in slightly saline soils (conductivity between 2 and 4 dS/m) (Katerji et al., 2001). The low ratio of lentil wild relative populations found to inhabit saline soils (one out of 624 populations) suggests that the sensitivity to soil salinity is widespread across the range of species comprising the genus *Lens*. Before the adaptation to soil salinity is tested in the selected population, it would be advisable to sample and assess the soil conductivity in that site to discard the possibility of an error in the digital cartography concerning this trait. Although the quality and accuracy of environmental information in digital cartography are continuously improving, we must be aware that the values assigned to geographical coordinates are the result of projections carried out through interpolation approaches and that the real values on site of the targeted variable may differ from the estimated ones. If the selected population is actually occurring in slightly saline soil, the transfer of the adaptive genes into cultivars will be hindered by the fact that *L. lamottei* is in the tertiary gene pool of cultivated lentils. Still, the potential benefits of accessing this wild species variability may be worth it (Van Der Maesen et al., 1988) and place the selected population as really valuable for pre-breeding purposes.

Finally, the environmental filtering method selected a relatively low number of wild lentil populations potentially tolerant to waterlogging (around 3.4% of total populations). Filtering criteria applied (bad drainage soils and humid sites according to the annual De Martonne aridity index) are in line with the main factors favoring waterlogging that affects lentil—type of soil and precipitation (Lake and Sadras, 2019). The low proportion of selected populations could agree with the high sensitivity of lentil to waterlogging in any of the vital stages, especially at the germination and vegetative stages (Materne et al., 2007; Nessa et al., 2007; Materne and Siddique, 2009; Malik et al., 2015), which would also be present in most of its wild relatives. A previous study on waterlogging tolerance carried out by Wiraguna et al. (2017) with cultivated accessions of lentils from major lentil-producing countries with different climates, found that only those from Bangladesh showed waterlogging tolerance at the germination stage. They argued that this could be due to its monsoonal climate (i.e., persistent rain depending on the season). The finding of this association supports our methodological approach in which we selected areas within the Mediterranean that relate to high precipitation and poor drainage.

### Calibration Method—Biotic Stress Analyses

According to the selected model (GBM, TSS value 0.828) we obtained a high percentage of wild populations potentially resistant to lentil rust (84.8%). This agrees with the findings of Singh et al. (2014) who experimentally assessed 405 wild lentil accessions and reported that many of the accessions were resistant or moderately resistant to rust. They identified two accessions, one accession of *L. nigricans* and another of *L. ervoides* that were especially valuable. These two species are the species in the subset selected by the calibration method applied in this study (nine *L. nigricans* populations and 21 *L. ervoides* populations in the subset of 30). Furthermore, they suggested Turkey and the Aegean area as priority areas for collecting, which are also represented in the subset of 30 populations for lentil rust resistance (22 populations in Turkey, Cyprus, and Greece). The remaining eight populations are located in other close areas: Croatia, Montenegro and Bosnia, and Herzogovina. Although *L. nigricans* and *L. ervoides* both belong to the secondary gene pool of lentils, the progress in the application of biotechnological techniques to obtain viable hybrids with cultivated lentils supports their use. For example, *L. ervoides* is an important source of variability to breed both biotic and abiotic stresses (Tullu et al., 2011, 2013). In addition, crosses made by Ladizinsky (1979) supported the utilization of *L. nigricans* almost at the same level of *L. culinaris* ssp. *orientalis* during breeding processes, although it is worth mentioning that *L. nigricans* is classified into two groups, one from South Europe and the other from the Middle East, the first one being cross-incompatible with cultivated lentils (Ladizinsky et al., 1983). The nine populations of *L. nigricans* in the reduced subset (30 populations) are found in the eastern Mediterranean which may facilitate their use in pre-breeding programs.

Abiotic and biotic stresses are likely to be interrelated and the interpretation and selection of models should follow a “judicious choice” (Challinor et al., 2009). In this sense, and given the similar TSS mean values, we considered variable importance as an additional criterion prior to the selection of the model to be projected. Further exploring the subset of 30 populations selected for rust, we found that all of them inhabit humid or sub-humid sites according to the De Martonne aridity index (see Supplementary Material 5, ecogeographical information section), which provide suitable conditions for the development of rust.

The inability to find a suitable model for broomrape resistance may be related to several reasons. In the first place, it may simply be explained by a lack of association between the tested ecogeographic variables and the pattern of resistance to broomrape. It may also be explained by the fact that the training set of the model was composed of lentil accessions that were mainly located in Spain and did not cover a wide enough environmental range. The incorporation of additional evaluation data on broomrape lentil resistance from wild populations in other countries could increase the quality of data and potentially improve predictive characterization results. We consider that the investment and enhancement in evaluation databases targeting broomrape resistance are of high interest and should be prioritized. Among others, resistance to broomrape is one of the traits lacking genetic variation in cultivated lentils (Sarker and Erskine, 2006) and the wider genetic diversity of wild lentil species could benefit its breeding. Another potential for improvement using the predictive characterization approach through the calibration method relies on the progress in the implementation of machine learning models. In this sense, the application of models that treat the response variable of biotic resistance on a quantitative basis may help improve the predictions.

## Conclusion

The interest in the incorporation of genetic diversity of wild lentils in pre-breeding and breeding programs is endorsed by recent studies targeting these species and the reported genetic diversity of adaptive value they possess (Ferguson and Robertson, 1999; Coyne and McGee, 2013; Kumar et al., 2014; Singh et al., 2014; Coyne et al., 2020; Id et al., 2020), to both biotic and abiotic stresses. The application of the predictive characterization techniques has successfully been applied in the search for traits of interest in other species (Bhullar et al., 2010; Bari et al., 2012, 2016; Khazaei et al., 2013; García Sánchez et al., 2019), and thus we consider our results of interest for the lentil plant breeding community. Furthermore, the generation of subsets explicitly pointing to small subsets of wild populations may put the focus on wild populations with higher possibilities of containing the desired trait other than randomly screening wild populations. Abiotic stresses, such as drought, salinity, and waterlogging, are major constraints in the production of lentils worldwide. Although there is presently a better understanding of adaptations to those conditions, research advances in this area are more limited than those related to damages by biotic interactions (Materne et al., 2007). Our results may contribute to facilitating access to lentil wild genetic resources, directing the exploration of novel germplasm for abiotic breeding purposes. Therefore, we suggest that the 13, one and 21 wild lentil populations predicted to be tolerant to drought, salinity, and waterlogging respectively, should be prioritized in trials to confirm such tolerances. In the same vein, we strongly recommend to sample and test for lentil rust resistance in the 30 populations of the generated reduced subset.

The application of predictive characterization methodologies is currently constrained to their use in particular cases followed by subsequent experimental validation. Further advances in the wider implementation of these techniques rely on the development of basic research that could provide a soundproof of concept. This would involve the implementation of experiments with integrative approaches combining genomic, environmental and phenotypic data that would provide insight into the mechanistic causes underlying the environment-phenotype associations.

## Data Availability Statement

The datasets presented in this study can be found in online repositories. The names of the repository/repositories and accession number(s) can be found below: https://github.com/MLRubioTeso/Searching-for-abiotic-tolerant-and-biotic-stress-resistant-wild-lentils-for-introgression-breeding-t.

## Author Contributions

MLRT and JI conceptualized and designed the research. MLRT, MP-Q, and JI agreed the methodology. DR performed the evaluation essays and generated the database with assessed lentil accessions used to calibrate the models. CL-R and MLRT curated the distribution data and generated the distribution wild lentil dataset. MLRT performed the analyses and wrote the original draft manuscript. All authors contributed to writing, reviewing and editing. JI supervised and managed the project and funding acquisition. All authors have read and agreed to the published version of the manuscript.

## Conflict of Interest

The authors declare that the research was conducted in the absence of any commercial or financial relationships that could be construed as a potential conflict of interest.

## Publisher’s Note

All claims expressed in this article are solely those of the authors and do not necessarily represent those of their affiliated organizations, or those of the publisher, the editors and the reviewers. Any product that may be evaluated in this article, or claim that may be made by its manufacturer, is not guaranteed or endorsed by the publisher.

## Footnotes

1
www.farmerspride.eu

2
www.genesys-pgr.org

3
https://drive.google.com/file/d/19ppFLclsF05ttpskjK8eILIJcXnlH0qc/view
