# Supplementary material for: Searching for Abiotic Tolerant and Biotic Stress Resistant Wild Lentils for Introgression Breeding Through Predictive Characterization
Source: Front Plant Sci. 2022 Mar 3;13:817849. doi: 10.3389/fpls.2022.817849 (PMC8928559; doi:10.3389/fpls.2022.817849)
Supplement: Supplementary file 2 [file Table_2.DOCX]

**Supplementary Material 2:** Script for the application of the Filtering Method of the Predictive Characterization

Script based and adapted from Van Etten, J.; Parra-Quijano, M.; Thormann, I.; Dias S.; Rubio Teso, M.L. & Iriondo, J. (2011): Basic script to execute a slightly modified version of the classical focused identification of germplasm strategy. Script developed during the Workshop on Predictive Characterization within the context of the PSG Secure Project: novel characterization of crop wild relatives and landraces as a basis for improved crop breeding ([www.pgrsecure.org](http://www.pgrsecure.org)). Workshop organized by Bioversity International in Rome (Italy) in December 2011.

*#Set working directory:*

setwd("C:/Users/MaríaLuisa/Universidad Rey Juan Carlos/Abiotic Filtering")

*#Call the dataset:*

Lens_DATASET<-read.delim("C:/Users/MaríaLuisa/Universidad Rey Juan Carlos/Abiotic Filtering /Lens_DATASET.txt")

*#Calculate frequency of ecogeographic categories in the dataset and add them to the ecogeographic name categories:*

FREQ_ECO<-table(Lens_DATASET$BGcat)

FREQ_ECO <- cbind(names(FREQ_ECO),as.vector(FREQ_ECO))

*#Order according to trait value: subset ordered according to the variable of our choice creating an index (Ar = aridity, variable = Annual De Martonne Index; Sa = salinity, variable = topsoil salinity (conductivity); Wa = waterlogging, variable = Soil Texture).*

Ar <- order(Lens_DATASET$DeMartonneANUAL)

Lens_DATASET_aridity <- Lens_DATASET[Ar,]

Sa <- order(Lens_DATASET$t_ece)

Lens_DATASETsalinity <- Lens_DATASET[Sa,]

Wa <- order(Lens_DATASET$TEX_CODE)

Lens_DATASETwaterlogging <- Lens_DATASET[Wa,]

*#Set the minimum number of records (n) from which you want the selection of the subset. “nc” is the number of ecogeographic units that have occurrences. “dim” function provides the dimension of the object (1:rows; 2:columns). “f” provides the frequency of each ecogeographical unit*

n <- 808 *# this is the total number of records*

nc <- dim(FREQ_ECO)[1]

f <- as.numeric(FREQ_ECO[,2])

*# calculate the proportional value of each frequency*

prop <- f/sum(f)

*#“even” object provides the minimum value between the proportional value and the even share. The second row is adjusted to sum a total frequency of 1.*

even <- pmin(prop,rep(1/nc, times=nc))

even <- even/sum(even)

*#“samples” is the number of samples that are to be selected from each ecogeographic unit. It combines the proportional and the even allocation approaches through the parameter "a". a=1 provides a complete proportional allocation, a=0 provides a truncated even allocation (even for those values where proportional is greater than even)*

a <- .5

samples <- (a*prop + (1-a)*even) * n

*# Give 0 value to samples if na is obtained from log(0) in the previous step*

samples[is.na(samples)] <- 0

*# round the values of samples and provide a value of at least 1 for those frequencies that are greater than zero*

samples <- pmax(round(samples),f>0)

*# Reorder the subset according to the variables in ascending (decreasing=FALSE) or descending (decreasing=TRUE) order.*

Ar <- order(Lens_DATASETaridity$DeMartonneANUAL,decreasing=FALSE)

Lens_DATASETaridity <- Lens_DATASET[Ar,]

Sa <- order(Lens_DATASETsalinity$t_ece,decreasing=TRUE)

Lens_DATASETsalinity <- Lens_DATASETsalinity[Sa,]

Wa <- order(Lens_DATASETwaterlogging$TEX_CODE,decreasing=TRUE)

Lens_DATASETwaterlogging <- Lens_DATASETwaterlogging[Wa,]

*# Remove records with NA values for the field BGcat (ecogeographic category).*

Lens_DATASETaridity <- Lens_DATASETaridity[!is.na(Lens_DATASET$BGcat),]

Lens_DATASETsalinity <- Lens_DATASETsalinity[!is.na(Lens_DATASET$BGcat),]

Lens_DATASETwaterlogging <- Lens_DATASETwaterlogging[!is.na(Lens_DATASET$BGcat),]

*# Remove records that have no data for the variable of interest*

Lens_DATASETaridiry <- subset(Lens_DATASETaridity, Lens_DATASETaridity$DeMartonneANUAL>0)

Lens_DATASETaridity <- subset(Lens_DATASETaridity, Lens_DATASETaridity$BGcat>0) *# We select BGcat (ecogeographic values) to use populations assigned to an ecogeographic unit of the ELC map, excluding those falling in category ‘0’ which lack information for some ecogeographic variables.*

Lens_DATASETsalinity <- subset(Lens_DATASETsalinity, !is.na(Lens_DATASETsalinity$t_ece))

Lens_DATASETsalinity <- subset(Lens_DATASETsalinity, Lens_DATASETsalinity$BGcat>0) *# We select BGcat (ecogeographic values) to use populations assigned to an ecogeographic unit of the ELC map, excluding those falling in category ‘0’ which lack information for some ecogeographic variables.*

Lens_DATASETwaterlogging <- subset(Lens_DATASETwaterlogging, Lens_DATASETwaterlogging$TEX_CODE>=1) *#TEX_CODE above 1 select soils with potentially bad water drainage (clay, silty-clay, sandy-clay, silty-clay-loam)*

Lens_DATASETwaterlogging <- subset(Lens_DATASETwaterlogging, Lens_DATASETwaterlogging$DeMartonneANUAL>0) *#By this selection we make sure to have values in the aridity field, that will be later needed.*

Lens_DATASETwaterlogging <- subset(Lens_DATASETwaterlogging, Lens_DATASETwaterlogging$BGcat>0) *# We select BGcat (ecogeographic values) to use populations assigned to an ecogeographic unit of the ELC map, excluding those falling in category ‘0’ which lack information for some ecogeographic variables.*

*# Create an ordered rank of the values of the ecogeographic categories:*

egregaridity <- unique(Lens_DATASETaridity$BGcat)

egregaridity <- egregaridity[order(egregaridity)]

egregsalinity <- unique(Lens_DATASETsalinity$BGcat)

egregsalinity <- egregsalinity[order(egregsalinity)]

egregwaterlogging <- unique(Lens_DATASETwaterlogging$BGcat)

egregwaterlogging <- egregwaterlogging[order(egregwaterlogging)]

*# Create a new object with empty values. Select one by one (through the loop) records from each ecogeographic unit. Add to the new object the number of records assigned to each ecogeographic unit*.

bssaridity <- NULL

for(j in 1:nc)

{

bsaridity <- subset(Lens_DATASETaridity, BGcat == egregaridity[j])

bssaridity <- rbind(bssaridity, bsaridity[1:samples[j],])

}

bssSalinity <- NULL

for(j in 1:nc)

{

bsSalinity <- subset(Lens_DATASETsalinity, BGcat == egregsalinity[j])

bssSalinity <- rbind(bssSalinity, bsSalinity[1:samples[j],])

}

bsswaterlogging <- NULL

for(j in 1:nc)

{

bswaterlogging <- subset(Lens_DATASETwaterlogging, BGcat == egregwaterlogging [j])

bsswaterlogging <- rbind(bsswaterlogging, bswaterlogging[1:samples[j],])

}

*# Order the new object according to the variable of interest. Reorder the ecogeographic set according to it.*

Ar <- order(bssaridity$DeMartonneANUAL,decreasing=FALSE)

bssaridity <- bssaridity[i,]

Sa <- order(bssSalinity$t_ece,decreasing=TRUE)

bssSalinity <- bssSalinity [Sa,]

Wa <- order(bsswaterlogging$DeMartonneANUAL,decreasing=TRUE) *#Populations inhabiting sites with low water drainage were selected before, we use De Martonne aridity index to select populations with high humidity levels.*

bsswaterlogging <- bsswaterlogging[Wa,]

*#Selection of subsets of populations according to desired values:*

*#****Aridity****: De Martonne classification (De Martonne, 1926): 0 < 5 extremely arid; ≥5 <10 – arid; ≥10 <20 – semiarid; ≥20 < 40 – subhumid; ≥40 < 60 - Humid; ≥60 perhumid).*

Lens_selection_aridity1<-subset(bss,DeMartonneANUAL<=10) *# X populations, arid classification*

Lens_selection_aridity2<-subset(bss,DeMartonneANUAL<=15) *# X populations, semi-arid classification*

Lens_selection_aridity3<-subset(bss,DeMartonneANUAL<=5) *#X populations, extremely arid classification*

*#Select best (most arid) 50 populations according to their Flowering De Martonne Index from the semiarid subset (Annual aridity).*

Lens_selection_flowering<-order(Lens_selection_aridity2$FloweringDeMartonne, decreasing=FALSE)

Lens_selection_flowering<-Lens_selection_flowering[1:50,]

*#* ***Salinity****: Topsoil Salinity above 4 dS/m is considered a threshold for crop salt tolerance (Panta et al. 2014; Walters, et al., 2010; Zörb et al. 2018). Topsoil salinity above 2 dS/m is considered slightly saline (Abrol et al., 1988)*

Lens_selection_salinity1<-subset(bssSalinity,t_ece>=4) # 0 records

Lens_selection_salinity2<-subset(bssSalinity,t_ece>=2) # 2 records

Lens_selection_salinityAll<-subset(bssSalinity,t_ece>0) # records

*# Selection of a second subset with the first best 50 populations according to the ordered (increasing) soil salinity levels in their sites*

Lens_selection_salinity50ordered<-order(Lens_selection_salinityAll$t_ece,decreasing=TRUE)

Lens_selection_salinity50ordered<- Lens_selection_salinity50ordered [1:50,]

*#* ***Waterlogging****: Populations inhabiting soils with bad water drainage previously selected. Selection of populations based on their aridity indexes (De Martonne classification (De Martonne, 1926): 0 < 5 extremely arid; ≥5 <10 – arid; ≥10 <20 – semiarid; ≥20 < 40 – subhumid; ≥40 < 60 - Humid; ≥60 perhumid)*.

Lens_selection_waterlogging1<-subset(bsswaterlogging,DeMartonneANUAL>=60) *#Perhumid populations*

Lens_selection_waterlogging2<-subset(bsswaaterlogging,DeMartonneANUAL>=40 & DeMartonneANUAL<60) *#Humid populations*

Lens_selection_waterlogging3<-(subset(bsswaterlogging,DeMartonneANUAL>=20 & DeMartonneANUAL<40)) *# Subhumid populations*

Lens_selection_waterlogging4<-subset(bsswaterlogging,DeMartonneANUAL>=20) *#All populations in subhumid areas or more humid areas.*

*# Selection of a subset with the first best 50 populations according to the humidity levels (decreasing order) in their sites from the fourth selection*

Lens_selection_waterlogging_50<-order(Lens_selection_waterlogging4$t_ece,decreasing=TRUE)

Lens_selection_waterlogging_50<-Lens_selection_waterlogging4[1:50,]

*#Write tables with the results:*

write.table(Lens_selection_aridity1, "Lens_ANNUAL_Aridity_Below10.txt", sep = "\t", col.names = NA, qmethod = "double")

write.table(Lens_selection_aridity2, "Lens _Annual_Aridity_Below15.txt", sep = "\t", col.names = NA, qmethod = "double")

write.table(Lens_selection_aridity3, "Lens _Annual_Aridity_Below5.txt", sep="\t", col.names = NA, qmethod = "double")

write.table(Lens_selection_flowering,"Lens_selection_Aridity_Flowering.txt", sep = "\t", col.names = NA, qmethod = "double")

write.table(Lens_selection_salinity1, "Lens_selection_Salinity_Above4.txt", sep = "\t", col.names = NA, qmethod = "double")

write.table(Lens_selection_salinity2, "Lens_selection_Salinity_Above2.txt", sep = "\t", col.names = NA, qmethod = "double")

write.table(Lens_selection_salinity50ordered,"Lens_selection_Salinity_50.txt", sep = "\t", col.names = NA, qmethod = "double")

write.table(Lens_selection_waterlogging1,"Lens_selection_Waterlogging_Perhumid.txt", sep = "\t", col.names = NA, qmethod = "double")

write.table(Lens_selection_waterlogging2,"Lens_selection_Waterlogging_Humid.txt", sep = "\t", col.names = NA, qmethod = "double")

write.table(Lens_selection_waterlogging3,"Lens_selection_Waterlogging_Subhumid.txt", sep = "\t", col.names = NA, qmethod = "double")

write.table(Lens_selection_waterlogging50,"Lens_selection_Waterlogging_Best50.txt", sep = "\t", col.names = NA, qmethod = "double")

***References:***

Abrol IP, Yadav JSP, Massoud FI. 1988. Saline soils and their management. Salt-Affected Soils and their Management. FAO Soils Bulletin 39. Rome, Italy

De Martonne E. 1926. Une nouvelle fonction climatologique: L’indice d’aridité. La Meteorologie.: 449–458

Panta S, Flowers T, Lane P, Doyle R, Haros G, Shabala S. 2014. Halophyte agriculture: Success stories. Environmental and Experimental Botany 107: 71–83

Walters, et al., 2010). Spatial prediction of topsoil salinity in the Chelif Valley, Algeria, using local ordinary kriging with local variograms versus whole-area variogram. Aust. J. Soil Res. 39, 259-272.

Zörb C, Geilfus CM, Dietz KJ. 2018. Salinity and crop yield. Plant Biology 21: 31–38.
